# Articaine versus Lidocaine in only buccal infiltration anesthesia for the extraction of mandibular anterior teeth. A prospective split-mouth randomized-controlled clinical study

**DOI:** 10.1186/s12903-023-03292-5

**Published:** 2023-08-28

**Authors:** Haytham Al-Mahalawy, Yehia El-Mahallawy, Hams H. Abdelrahman, Shaimaa Mohsen Refahee

**Affiliations:** 1 Oral and Maxillofacial Surgery Department, Faculty of Dentistry, Fayoum University, Fayoum, Egypt; 2 Oral and Maxillofacial Surgery Department, Faculty of Dentistry, Alexandria University, Alexandria, Egypt; 3 Dental Public Health and Pediatric Dentistry Department, Faculty of Dentistry, Alexandria University, Alexandria, Egypt; 4 Oral & Maxillofacial Surgery Department, Faculty of Dentistry, Fayoum University, Fayoum, Egypt

**Keywords:** Articaine, Lidocaine, Only buccal infiltration, Supplementary lingual injection, Mandibular anterior teeth

## Abstract

**Objective:**

To investigate the effectiveness of a single labial infiltration of 4% articaine versus 2% lidocaine for the extraction of mandibular anterior teeth without an additional lingual injection.

**Patients and methods:**

A prospective, randomized-controlled, split-mouth clinical study was implemented. Healthy adult patients seeking bilateral extraction of mandibular anterior teeth were included in this study. Teeth extractions were randomly assigned to two equal groups, where one mandibular anterior tooth was extracted using a solitary labial infiltration of either 4% articaine (the study group) or 2% lidocaine (the control group). After 14 days, the other mandibular anterior tooth was extracted using the other local anesthetic agent. The selection of the anesthetic agent injected in the first session was done in a randomized fashion. After 5 min of local anesthetic injection, the tooth was extracted, and each patient was asked to record the intensity of the extraction pain using the Visual Analogue Scale (VAS).

**Results:**

Thirty-one patients were included in the study. The efficacy of a single labial injection for mandibular anterior teeth extraction was established by the fact that none of the patients in the study or control group required re-administration of local anesthesia. The mean VAS for pain control during tooth extraction was 1.16 ± 0.93 for the articaine group and 1.71 ± 0.90 for the lidocaine group. The pain score showed a statistically significant decrease in the articaine group compared to that in the lidocaine group (*P* = 0.017).

**Conclusion:**

Although the anesthetic effects of only buccal infiltration of 4% articaine and 2% lidocaine for extraction of mandibular anterior teeth were comparable, the use of 4% articaine would have more effective and predictable outcomes.

**ClinicalTrials.org:**

(ID: NCT05223075) 3/2/2022.

**Supplementary Information:**

The online version contains supplementary material available at 10.1186/s12903-023-03292-5.

## Introduction

Profound anesthesia is the most important aspects to achieve successful dentoalveolar extraction. Several anesthetic drugs and techniques are available to attain this goal. The Inferior alveolar nerve block (IANB) is the main technique used to anesthetize the mandibular teeth, but it is more effective in the molar and premolar areas than in the anterior teeth. The IANB has a significant failure rate, ranging from 31% to 41% in mandibular second and first molars to 42%, 38%, and 46% in second and first premolars and canines, respectively, and 81% in lateral incisors [1].

The incomplete anesthesia of IANB in the anterior region may be attributed to the overlapping of the mylohyoid fibers and/ or the contralateral inferior alveolar nerve fibers in this area [2, 3]. Furthermore, this compromised anesthesia could be explained by the central core theory. The molar teeth are supplied by mantle fibers (nerves on the outsideof the nerve bundle), whereas the incisor teeth are supplied by core fibers (fibers on the interior). As a result, a local anesthetic solution applied near the inferior alveolar nerve may spread and block the outermost fibers but not those more centrally located. This results in inadequate mandibular anesthesia for the anterior teeth [4]. Accordingly, the infiltration technique is more suitable in this area than the IANB [2, 3].

According to the anatomical consideration of the mandibular anterior area, where the labial cortical plate of bone is thin enough to the degree that allows infiltration technique to be effective, labial infiltration with an additional lingual injection is required before tooth extraction to achieve profound anesthesia [1].

The main concept in local anesthesia is to achieve profound anesthesia with the least amount of medication and the least number of tissue penetrations to decrease the possibility of complications and the patient’s anxiety [1, 5–7].

Different local anesthetic agents were introduced into the market and approved for their efficacy. One of them is articaine, which is considered a member of the amide family with a thiophene ring and ester linkage. The increased liposolubility of articaine makes it 1.5 times the potency and 0.6 times the toxicity of lidocaine [1]. In addition, articaine is characterized by its prominent bony and soft tissue diffusion properties, owing to the presence of the thiophene ring [8, 9]. Furthermore, articaine’s rapid metabolism permits its use in higher concentrations with lower systemic toxicity, resulting in a safer anesthetic solution with a longer duration of action [1]. Despite the abovementioned clinical advantages of articaine over the other local anesthetic agents, articaine has been claimed to be associated with a higher risk of prolonged soft tissue anesthesia or paresthesia, especially when used for inferior alveolar nerve blocks [10, 11].

Several studies have approved the diffusion power of articaine, where it was concluded that labial infiltration of 4% articaine was enough to extract maxillary teeth without an additional palatal injection [5, 7]. Furthermore, a clinical trial conducted in 2016 concluded that the combination of buccal and lingual infiltrations with 4% articaine was adequate for the extraction of mandibular first molars with no need for nerve block injection [12]. Unlike in the maxillary teeth, there is a lack of consensus regarding the solitary use of labial infiltration in the extraction of mandibular anterior teeth, as several studies stated that the combined buccal and lingual infiltrations improve the effect of pulpal anesthesia in the mandibular anterior area more than buccal infiltration alone [13, 14]. Accordingly, there is a lack of studies regarding the diffusion power of 4% articaine in the mandibular anterior area and its efficacy for tooth extraction as only labial infiltration without supplementary lingual injection, where the least amount of anesthesia will decrease the possible complication and the least number of tissue penetrations will decrease the patient’s anxiety, Therefore, the authors suggested that solitary labial articaine infiltration could be more efficient than solitary labial lidocaine infiltration.

The aim of the present study was to compare the effectiveness of a solitary labial infiltration of 4% articaine with that of 2% lidocaine on intraoperative pain control during the extraction of mandibular anterior teeth without the necessity for additional lingual injection.

## Patients and methods

**Fig. 1:**
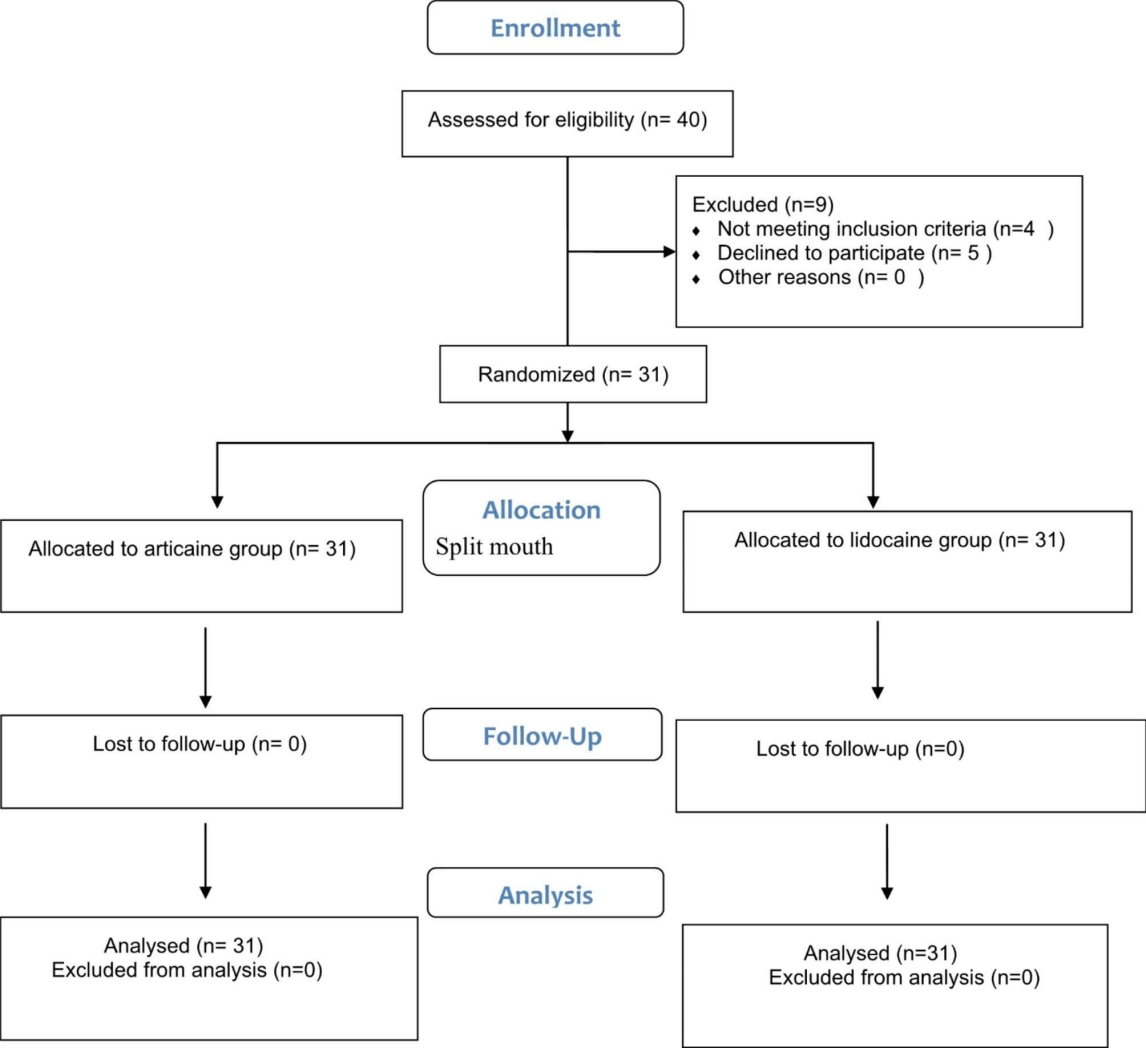
Consort flow chart

This study was conducted in the Oral and Maxillofacial Surgery Department, Faculty of Dentistry, according to the principles of Helsinki, and the CONSORT guidelines (Fig. 1) [15, 16]. The study protocol was approved by the ethics committee of Fayoum University (EC 2221) and was registered on ClinicalTrials.org (ID: NCT05223075). All patients in the study provided their informed consent after clarifying the practices and their harm to them.

### Sample size calculation

A power analysis was planned to have suitable power to apply a two-sided statistical test of the null hypothesis that there is no difference would be found between the two groups. The calculated sample size (n) was 31 patients at an alpha level of (0.05), power = 80%, and an effect size (d) of (0.52) depending on the results of an earlier study [7]. The sample size was calculated by G*Power version 3.1.9.7.

### Study design

This was a prospective, randomized (1:1), controlled, double-blinded, split-mouth trial, where the effectiveness of a solitary labial infiltration of 4% articaine with 1:100,000 epinephrine was evaluated and compared to that of 2% lidocaine with 1:100,000 epinephrine for the extraction of mandibular anterior teeth without an additional lingual injection.

### Inclusion and exclusion criteria

Participants were enrolled from those referred to the clinics of the Faculty of Dentistry, Fayoum University, for the extraction of bilateral mandibular anterior teeth during the period between November 2022 and March 2023. Adult patients, with no gender preference, no relevant medical history, and seeking bilateral, non-surgical extraction of permanent mandibular anterior teeth (either symmetrical or asymmetrical), were included in the study. On the other hand, all patients with highly mobile teeth (Grade 2–3), infection at the needle pathway, sensitivity to lidocaine or articaine local anesthetics, or analgesics use the day before the procedure were excluded.

### Data collection

A ‘split-mouth’ design was applied, where one mandibular anterior tooth was randomly extracted using a solitary labial infiltration of either 4% articaine (study group) or 2% lidocaine (control group). After 14 days, the other cross-arch mandibular anterior tooth was extracted using the other local anesthetic agent. The group allocation for the choice of the local anesthetic agent used in the first extraction session was done randomly using a computer-generated randomization list. In an attempt to blind the content of the anesthetic cartridges from the operator, identical cartridges were used and shielded with opaque adhesive. The surgeon, patients, and assessor were blinded from the type of local anesthetic used.

#### Intervention steps

For all the participant in this study, local anesthesia was delivered by one surgeon, and the extraction was completed by another surgeon. The surgeon who performed the extraction was not aware of the utilized local anesthetic agent. A standardized labial supra-periosteal infiltration technique was used for both groups by using a 27-gauge short needle that was inserted at a 45° angle with the long axis of the tooth and the injection rate was kept at 60 s. The study group received a single labial infiltration of 1.7ml of 4% articaine HCL with 1:100,000 epinephrine (Septocaine, Septodont, New Castle, Del.). The control group received a single labial infiltration of 1.8 ml of 2% lidocaine HCL with 1:100,000 epinephrine (Xylocaine, AstraZeneca, York, Pa.). The extraction was performed five minutes after the local anesthetic injection.

#### Outcome assessment

The primary outcome was the evaluation of pain control during extraction of mandibular anterior teeth after the administration of only labial infiltration of either 2% lidocaine or 4% articaine local anesthesia without an additional lingual injection. The recorded pain value was assessed by a 10-points Visual Analogue Scale (VAS / cm), with a zero end that denotes no pain and a 10-end that represents the maximum pain sensation. Furthermore, the necessity for re-administering anesthesia was considered a failure. A descriptive analysis of the demographic variables, tooth type, and the causes of extraction was performed.

### Statistical analysis

Normality was tested using the Shapiro-Wilk test and Q-Q plots. Values were not normally distributed. Therefore, median, Inter Quartile Range (IQR), minimum, and maximum were mainly used to present the data in addition to mean and standard deviation. Differences between groups were analyzed using the Wilcoxon sign rank test, while comparisons between groups regarding VAS based on tooth type were performed using the Mann-Whitney U test. All tests were two-tailed, and the significance level was set at a *P* value ≤ 0.05. Data were analyzed using IBM SPSS Statistics for Windows, Version 24.0. Armonk, NY: IBM Corp.

## Results

Thirty-one patients (10 (32.3%) females and 21 (67.7%) males) were included in this study, with ages that ranged between 30 and 70 (average 59.8) years old. The teeth are extracted due to prosthetic causes, deep caries, and periodontitis. The canine (n = 25, 40.32%) was the most involved tooth in the study, followed by the lateral incisor (n = 20, 32.26%), and the central incisor (n = 17, 27.42%) (Table 1). The efficacy of a single labial infiltration injection for mandibular anterior teeth extraction was established by the fact that none of the patients in the study or control group required re-administration of local anesthesia. Furthermore, the safety was also established, as none of the patients in both groups reported any post-operative anesthetic complications. The mean recorded VAS for pain control during extraction was 1.16 ± 0.93 in the articaine group and 1.71 ± 0.90 in the lidocaine group. The pain score showed a statistically significant decrease in the articaine group compared to that in the lidocaine group (*P* = 0.017) (Table 2). Regarding the comparison of VAS during extraction between groups based on tooth type, there was no significant difference between articaine and lidocaine in pain score following the extraction of the mandibular central and lateral incisors, whereas there was a significant difference in favor of articaine following the extraction of the mandibular canines (Table 2).

**Table 1:** Participants’ baseline characteristics

		N = 31 patients, 62 sides.
Age: Mean ± SD		59.87 ± 8.97
Gender: n (%)	Female	10 (32.3%)
	Male	21 (67.7%)
Tooth type: n (%)	Centrals	17 (27.42%)
	Laterals	20 (32.26)
	Canines	25 (40.32%)

**Table 2:** Comparison of (VAS) following extraction between the study and control groups

Tooth type		VAS Score / Cm.		*p* value
		Study group (n = 31 sides)	Control group (n = 31 sides)	
Centrals	Mean ± SD	1.38 (1.19)	1.67 (1.00)	0.650
	Median (IQR)	2.00 (2.00)	2.00 (2.00)	
	Min – Max	0.00–3.00	0.00–3.00	
Laterals	Mean ± SD	1.10 (0.74)	1.50 (0.53)	0.208
	Median (IQR)	1.00 (1.00)	1.50 (1.00)	
	Min – Max	0.00–2.00	1.00–2.00	
Canines	Mean ± SD	1.08 (0.95)	1.91 (1.14)	0.049*
	Median (IQR)	1.00 (2.00)	2.00 (2.00)	
	Min – Max	0.00–3.00	0.00–3.00	
				***p*** **value**
Total	Mean ± SD	1.16 (0.93)	1.71 (0.90)	0.017*
	Median (IQR)	1.00 (2.00)	2.00 (1.00)	
	Min – Max	0.00–3.00	0.00–3.00	

*Statistically significant at *p* value ≤ 0.05

## Discussion

The goal of this study was to assess the effectiveness of using either 4% articaine or 2% lidocaine as a single labial infiltration for intraoperative pain management during mandibular anterior teeth extraction without an additional lingual injection. This will aid in the proper selection of the ideal local anesthetic agent that will achieve a higher and more predictable success rate. This goal was determined clinically using the pain control effectiveness during extraction, which was assessed subjectively using the VAS following extraction.

Results of this study proved the effectiveness of solitary labial infiltration anesthesia without an additional lingual injection for the extraction of mandibular anterior teeth, where all the subjects in the study and control groups tolerated the extraction without the need for re-administration of additional local anesthesia. These findings are in agreement with those of Ege & Demirkol, where they concluded that the extraction of mandibular incisors and premolars could be achieved with a single buccal infiltration of 2% lidocaine [6].

Results of this study have proved the superiority of articaine over lidocaine for the only buccal infiltration anesthesia in the anterior mandibular area where the pain score during extraction showed a statistically significant decrease in the articaine group compared to that of the lidocaine group. This superiority has been proven in several previous clinical studies and systematic reviews comparing the two agents in other intraoral locations using various infiltration and block techniques [5, 7, 17, 18]. This superiority could be attributed to the unique chemical characteristic features of articaine. It is a modification of the typical amide local anesthetic solution group, where the benzene (aniline) ring is replaced by a sulfur-containing thiophene ring, with an elimination serum half-life of 20–30 min. The lipophilic thiophene ring characterizes articaine with higher rates of lipid solubility than its amide kin, which in consequence gives the solution higher potency and diffusion ability [17]. This feature gives the articaine the upper hand in infiltration injection in the anterior mandibular region owing to the articaine’s unique diffusion characteristics and the reduced thickness of the mandibular bone in this area. Another articaine tissue/bone penetration mechanism may be explained by its ability to form an intramolecular hydrogen bond [19].

Results of this study regarding the comparison of the mean VAS between groups during extraction based on tooth type showed that there was no significant difference between the articaine and lidocaine in pain score during extraction of the mandibular central and lateral incisors, while there was a significant difference in favor of articaine during the extraction of mandibular canines. This finding could be attributed to the increased buccolingual thickness of bone in the canine region, which requires a local anesthetic agent with higher bony and soft tissue diffusion properties [9, 19].

In this study, the safety of articaine was established, where none of the patients reported any post-operative anesthetic complications. These results could be attributed to the technique utilized in this study, which is a supra-periosteal infiltration of the dental branches of the mandibular incisive nerve with no affection of the mental nerve. These findings support the use of articaine as a solitary buccal infiltration technique in the mandibular anterior region instead of inferior alveolar or mental nerve blocks, as no incidence of paresthesia has been reported in the literature when using the infiltration technique [11].

Attempts were taken in this study to reduce the confounding factors, as the study outcomes are mainly subjective. Individuals having a local infection, neurological disorder, or taking analgesics one day before from extraction were excluded to avoid any effect on the pain perception. Furthermore, the study adopted a split-mouth design, and the participants and the operator were blinded from the utilized type of local anesthesia.

However, the study has a limitation that includes the sequential administration of anesthesia, which could increase the possibility of period effects, and the inability to eliminate the patient’s psychological pain tolerance during the second injection. This could result in potentially biased pain perception. Further clinical studies, confined to the posterior region of the mandible and addressing the same concept of only buccal infiltration without lingual injection, may be required to assess the effect of increased bucco-lingual bone width on the effectiveness of articaine penetration ability in this region.

## Conclusion

The favorable clinical outcomes achieved in this study demonstrate that the use of a solitary labial infiltration during the extraction of mandibular anterior teeth without an additional lingual injection is an effective anastatic option for intraoperative pain control. Although the anesthetic effects of a solitary buccal infiltration of articaine or lidocaine for the extraction of mandibular anterior teeth were comparable, the use of 4% articaine would have more effective and predictable outcomes, especially in the canine region, as it provides an improved ability to diffuse through soft and hard tissues.

### Electronic supplementary material

Below is the link to the electronic supplementary material.

Supplementary Material 1

## Data Availability

The datasets of the study are available as supplementary materials.

## Abbreviations

IANB: inferior alveolar nerve block
IQR: Inter Quartile Range
VAS: visual analogue scale
